# Detection of cancer stem cells by immunohistochemistry of canine mammary neoplasias

**DOI:** 10.1186/1753-6561-7-S2-P28

**Published:** 2013-04-04

**Authors:** Geórgia Modé Magalhães, Erika M Terra, Rosemeri O Vasconcelos, Mayara C Rosolem, Márcio B Bandarra, Pâmela RR Moreira, Antonio C Alessi

**Affiliations:** 1 Department of Veterinary Pathology, UNESP, Jaboticabal, SP, Brazil; 2 Department of Veterinary Clinics and Surgery, UNESP, Jaboticabal, SP, Brazil

## Background

Canine mammary neoplasias are very common and, in Brazil, 70% are considered malignant. Cancer stem cells (CSCs) are able to self-renew and have abilities to form metastases. In canine mammary tumors these CSCs were isolated by flow cytometry by means of the surface markers CD44+/CD24-. The aims of this study was detect these CSCs by immunohistochemical reactions and correlate them with degrees of canine mammary neoplasias.

## Patients and methods

A total of 130 breast cancer samples were selected from the Unesp - Department of Pathology and classified according to [1]. These samples were composed of adenomas; metastasis, solid carcinoma grades II and III; tubular, papillar and mixed tumor carcinoma grades I, II and III. Immunohistochemical was performed with antibodies CD44 and CD24.

## Results

A regression line by Pearson correlation test was performed. The value which CD44 is positive and CD24 becomes zero is 46.75%. From 130 samples, 40 showed the phenotype CD44+/CD24-Thirty seven was metastasis, grades II and III. The immunostainings for CD44 and CD24 antibodies were respectively 62.2% and 0%. In the literature, grade III are more correlated with CD44 than CD24. In studies by flow cytometry authors found percentages of CSCs similar to those found in this work.

## Conclusion

The immunohistochemistry showed to be a reliable technique for detection CSCs in canine mammary neoplasias and correlates with grades II and III (poor prognosis).

## Financial support

CNPq.
